# Supplementary material for: Polariton design and modulation via van der Waals/doped semiconductor heterostructures
Source: Nat Commun. 2023 Dec 2;14:7965. doi: 10.1038/s41467-023-43414-9 (PMC10693602; doi:10.1038/s41467-023-43414-9)
Supplement: Supplementary file 1 — Supplementary Information [file 41467_2023_43414_MOESM1_ESM.pdf]

Supplementary information:

Polariton design and modulation via van der Waals/doped semiconductor heterostructures

Mingze He[1,9], Joseph R. Matson[2,9], Mingyu Yu[3], Angela Cleri[4], Sai S. Sunku[5], Eli Janzen[6], Stefan Mastel[7], Thomas G. Folland[8], James H Edgar[6], D. N. Basov[5], Jon-Paul Maria[4], Stephanie Law[3,4], Joshua D. Caldwell[*,1, 2]

1. Department of Mechanical Engineering, Vanderbilt University, Nashville, Tennessee, 37240, USA

2. Interdisciplinary Materials Science Program, Vanderbilt University, Nashville, Tennessee, 37240, USA

3. Department of Materials Science and Engineering, University of Delaware, Newark, Delaware, 19716, USA

4. Department of Materials Science and Engineering, The Pennsylvania State University, University Park, Pennsylvania, 16802, USA

5. Department of Physics, Columbia University, New York, NY 10027, USA

6. Tim Taylor Department of Chemical Engineering, Kansas State University, Manhattan, Kansas, 66506, USA

7. Attocube Systems AG, Haar (Munich), 8550, Germany

8. Department of Physics and Astronomy, The University of Iowa, Iowa City, Iowa, 52242, USA

9. These authors contributed equally: Mingze He, Joseph R. Matson

[*] josh.caldwell@vanderbilt.edu

Contents

Supplementary information: 1

Polariton design and modulation via van der Waals/doped semiconductor heterostructures 1

Note 1. Example of HPhP wavevector extraction 2

Note 2. Transitional frequency 2

Note 3. Surface roughness of doped semiconductors 3

Note 4. hBN on in-plane varying doped semiconductor 4

Note 5. Thickness dependence of the modal order transition 5

Note 6. Intriguing behaviors of HPhPs around transitional point 7

Note 7. Data analysis on the transitional HPhP sample 8

Note 8. Sensitivity to the local environment around modal order transition 9

Note 9. Loss associated with the tuned HPhPs 9

Note 10. Dispersion of HPhPs at ultrafast time scale 11
Note 11. Data process of ultrafast measurements 11
Note 12. The collective effect of HPhPs in the temporal domain 12

Note 1. Example of HPhP wavevector extraction

Here we provide two examples of HPhP wavevector extraction from s-SNOM data. We first extracted line profiles from s-SNOM images (**Supplementary Figure 1a, d**). Then the line profiles were extended with "zero filling", as shown in **Supplementary Figure 1b**. With the zero filled line profiles, fast Fourier transform (FFT) was performed, followed with peak fitting to extract the wavevector information (**Supplementary Figure 1c**). After that, the wavevector of HPhPs (k_{HPhP}) are acquired. Data analysis of two samples at 1500 cm^{-1} are presented in **Supplementary Figure 1**. Note that for the transitional HPhP sample, the loss is so high that no fringes are observed, and we employed a different strategy to do the data analysis (**Supplementary Note 7**).

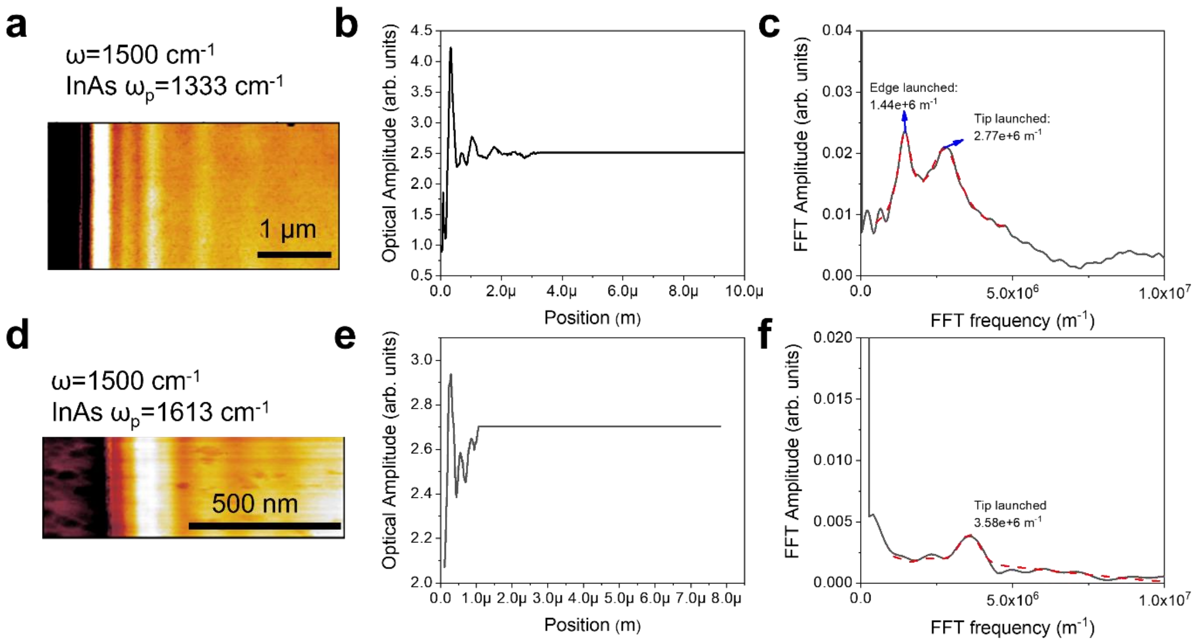

Supplementary Figure 1. Wavevector analysis from s-SNOM images. (a) s-SNOM image. (b) Line-profile extracted from panel a. (c) FFT of panel b, and peak fitting conducted on the FFT data to extract wavevector. (d-f) Same process, but for a different sample.

Note 2. Transitional frequency

Here we discuss where the modal order transition happens, and the working frequency is 1500 cm^{-1} to be consistent with **Fig. 2** and **Fig. 3a** in the main text. We first discuss whether the loss of the substrate will

change the transitional frequency, and we calculate the normalized k_{HPhP} with different imaginary parts associated with the real part, as shown in **Supplementary Figure 2a**. Despite different losses, the transitional frequencies of all four cases are identical, and they all happen where the $\mathrm{Re}(\varepsilon_s)$ is -1. Additionally, we assign the top environment ($\varepsilon_{superstrate}$) with different permittivity values, and we analyze if the transitional frequency changes. In this case, we find the transition always happens where $\mathrm{Re}(\varepsilon_s) + \varepsilon_{superstrate}=0$ (**Supplementary Figure 2b**). This also explains why the HPhP system is sensitive to the surrounding material index around the transitional point (**Supplementary Note 4**), as the transitional frequency is modified, which leads to pronounced dispersion change.

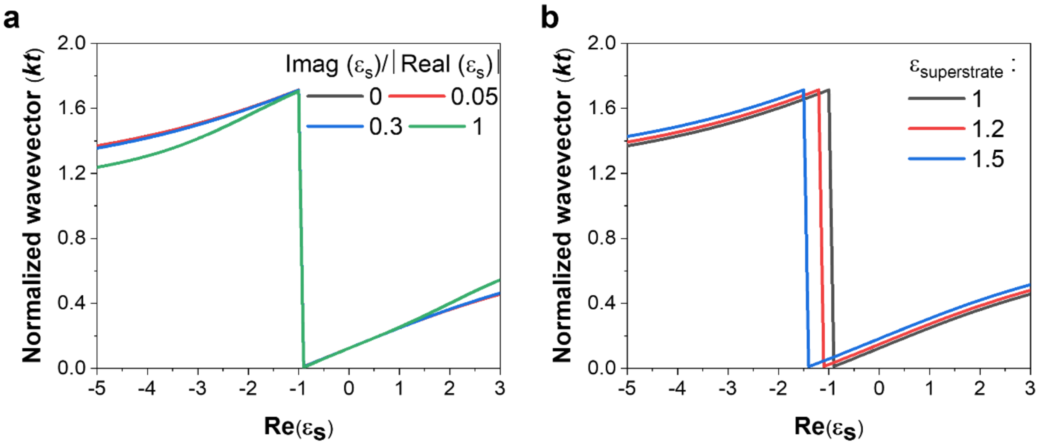

Supplementary Figure 2. HPhP modal transition frequency dependence. (a) HPhP modal order transition always happens at $\mathrm{Re}(\varepsilon_s) = -1$, despite with differently assigned loss of the substrate. (b) HPhP modal order transition changes with varying $\varepsilon_{superstrate}$.

Note 3. Surface roughness of doped semiconductors

To extract the surface roughness, we employed AFM measurements on two representative doped semiconductor samples, as shown in **Supplementary Figure 3**. Since InAs are grown by molecular beam epitaxy (MBE), they feature very low surface roughness (~ 0.3 nm). CdO samples are fabricated on high-power impulse magnetron sputtering, and the surface roughness is ~0.5 nm.

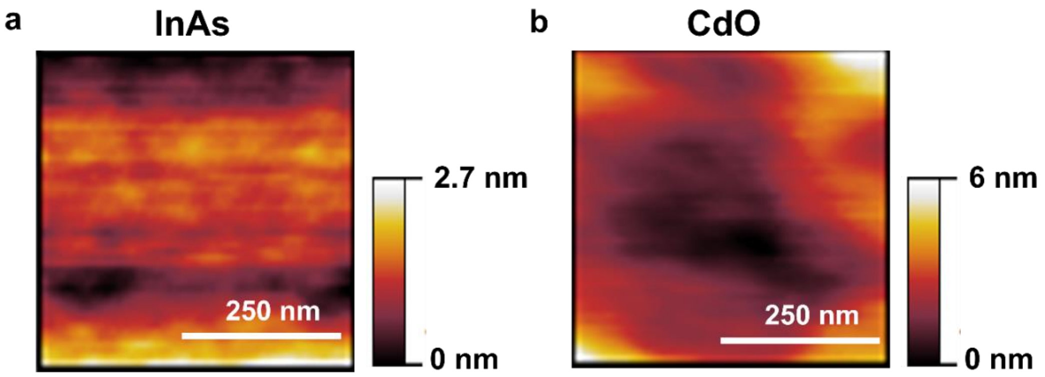

Supplementary Figure 3. Surface roughness measurements on InAs and CdO, respectively.

Note 4. hBN on in-plane varying doped semiconductor

Here we show that HPhP wavevectors supported in a single pristine hBN can be manipulated by controlling the carrier density of the semiconductor in contact with the material, in this case CdO. The CdO matrix is unintentionally doped (UID), i.e., it contains oxygen vacancies during the growth process, and the estimated plasma frequency is ~1600 cm^{-1}. Then approximately half of the sample is then exposed to 1 MeV Ar^{+} ion irradiation to induce additional native donor defects in the material and locally increase the carrier concentration, thereby shifting the plasma frequency to about ~3000 cm^{-1}. This produces a lateral transition to a higher plasma frequency as defined by the mask edge with no discernable change in surface microstructure. An hBN flake is then exfoliated over this interface, and dramatically different HPhP wavelengths are observed (**Supplementary Figure 4a**). Quantitatively, the wavevector difference over the two regions is ~2 times, and they are plotted over TMM calculations, with the data from irradiated and UID regions shown as triangles and rectangles, respectively (**Supplementary Figure 4b, c**). Note that the carrier concentration transition is not perfectly abrupt across the defined regions because the photoresist mask walls are not perfectly straight, creating a gradient in irradiation damage near the interface. The surface roughness in the previously masked regions is higher (~ 5 nm RMS) due to residual photoresist residue that could not be 100% removed. Irradiation causes unintended resist hardening that is difficult to avoid. Because the surface roughness of pristine CdO is ~0.5 nm (**Supplementary Note 3**), we know that the surface roughness of this type of structure can be reduced to sub-nanometer with optimized fabrication. Regardless, this preliminary demonstration opens up possibilities to manipulate HPhPs with semiconductor structures with carrier concentration variations across lateral interface geometries limited only by lithography limitations.

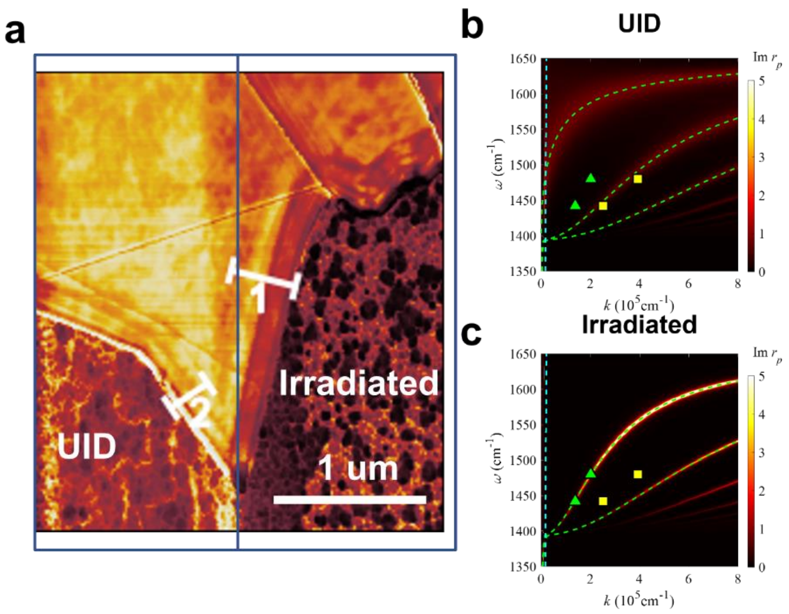

Supplementary Figure 4. HPhPs manipulated by in-plane doped semiconductors. (a) S-SNOM image of hBN over in-plane irradiated CdO at 1442 cm^{-1}. The two lines denote where line profiles are taken to extract wavevectors. Note that the high surface roughness here (~5 nm) is caused by photoresist residues, which increases polariton scattering. We expect the surface roughness can be reduced to ~1 nm upon further procedure optimization, as the surface roughness of pristine CdO is ~0.5 nm **(Supplementary Note 3**).

Note 5. Thickness dependence of the modal order transition

Here we discuss the thickness dependence of the modal order transition. Based on the analytical solution (assuming $k_{HPhPs} >> k_0$), the modal order transition should not be thickness dependent. The analytical solution for the HPhP wavevector is:

$$k(\omega) = k' + ik'' = -\frac{\psi}{d}\left[\text{atan}\left(\frac{\varepsilon_o}{\varepsilon_t\psi}\right) + \text{atan}\left(\frac{\varepsilon_s(\omega)}{\varepsilon_t\psi}\right) + \pi l\right], \quad \psi = -i\sqrt{\frac{\varepsilon_z}{\varepsilon_t}} \qquad \text{Eq. S1}$$

which can be rewritten as:

$$k(\omega)d = -\psi\left[\text{atan}\left(\frac{\varepsilon_o}{\varepsilon_t\psi}\right) + \text{atan}\left(\frac{\varepsilon_s(\omega)}{\varepsilon_t\psi}\right) + \pi l\right], \quad \psi = -i\sqrt{\frac{\varepsilon_z}{\varepsilon_t}} \qquad \text{Eq. S2}$$

and the transition happens for hBN of any thickness when

$$\text{atan}\left(\frac{\varepsilon_o}{\varepsilon_t\psi}\right) + \text{atan}\left(\frac{\varepsilon_s(\omega_{tran})}{\varepsilon_t\psi}\right) = 0 \qquad \text{Eq. S3}$$

where l is a non-negative integer representing the HPhP mode order (0,1,2…). d represents the hBN thickness, ε_o and ε_s the complex dielectric functions of the superstrate (air here) and the substrate, respectively, and ε_t and ε_z are dielectric functions of hBN along the in and out of plane axes. The 0-order mode is no longer supported when $\text{atan}\left(\frac{\varepsilon_o}{\varepsilon_t\psi}\right) + \text{atan}\left(\frac{\varepsilon_s}{\varepsilon_t\psi}\right)$ is negative, which is referred to as modal order transition in the main text. We note that as the wavevector is both frequency and thickness dependent in hyperbolic media, solving Eq. S2 for the $k(\omega)d$ product normalizes the thickness dependence of the

wavevector and thus, generalizes the solution to arbitrary thicknesses of hBN within the thin film limit (see further discussions on this limit below).

For thin hBN, the above discussion is precise as Eq. S2 is accurate, since the HPhP is very dispersive and the wavevectors are generally much larger than k_0., as validated by experiments (**Supplementary Figure 6**). However, for thick hBN, the wavevectors are reduced and the accuracy of the analytical solution degrades in a wider frequency range, affecting the modal order transition criteria. For thick hBN, e.g., 300 nm, at a frequency that is higher than ω_{tran} in Eq. S4, the k_{HPhP} becomes large enough to satisfy the large wavevector assumption. Therefore, the transition frequency is higher for thick hBN, as shown in **Supplementary Figure 5**.

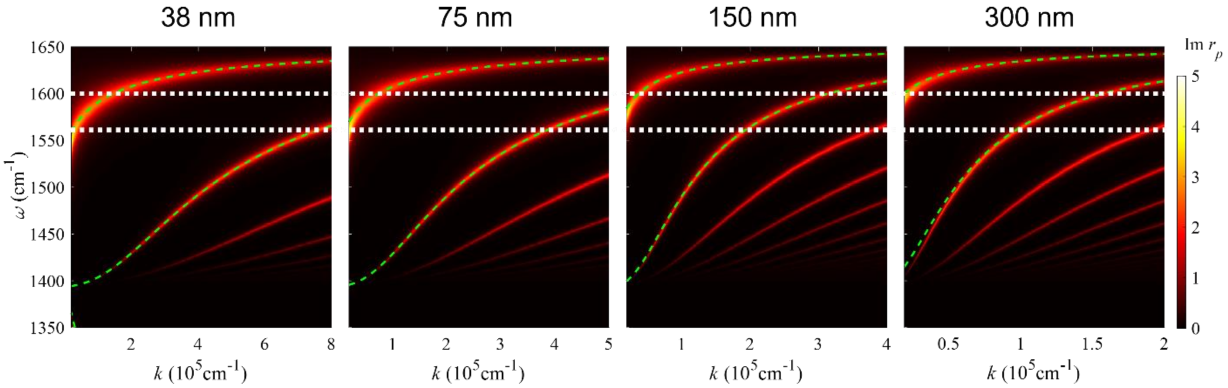

Supplementary Figure 5. The dispersion plot of hBN/InAs (ω_p=1612 cm^{-1}) with different thicknesses. The InAs ω_p is 1612 cm^{-1}. The reference lines denote the range of transition frequencies with different hBN thicknesses. Note that the transition frequency does not change much with hBN thinner than 150 nm.

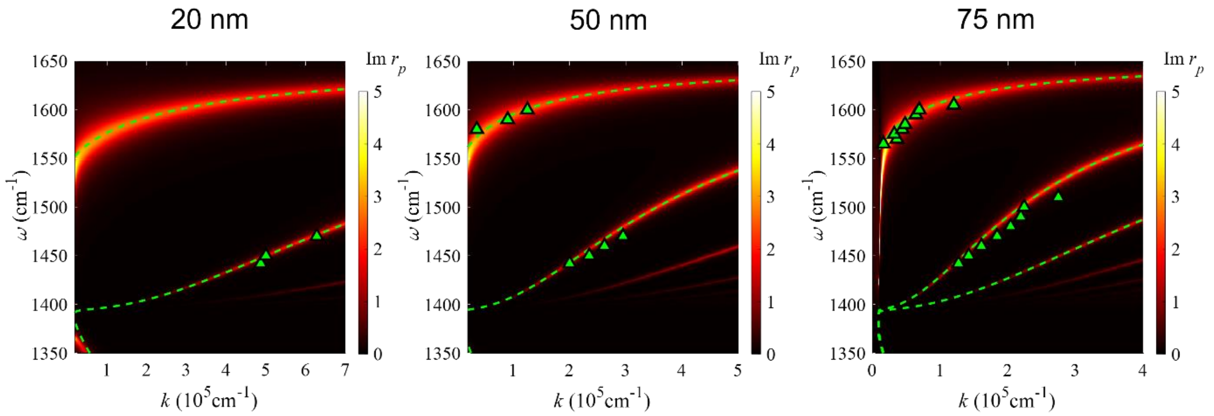

Supplementary Figure 6. The dispersion plot of hBN/InAs with different hBN thicknesses (labeled in the figure). The InAs ω_p is 1612 cm^{-1} in all three plots (also the same as **Supplementary Figure 5**), validating the calculations. The experimental data are plotted as green triangles and the analytical solution is plotted as dashed curves.

Note 6. Intriguing behaviors of HPhPs around transitional point

Here we provide two examples of HPhP wavevector extraction from s-SNOM data, one in the frequency domain (far-field resonators) and the other one in the real space (polariton propagations).

For far-field resonator designs, e.g., a sub-diffractional grating, the resonance frequency can be approximated as where the wavevector matches the required value. Here we exemplify a simple case: 450 nm wide (900 nm period) sub-diffractional grating structure, where a wavevector of n times ~0.4×10^{5} cm^{-1} is required. In a HPhP system with modal transition, there will be two cross-points (**Supplementary Figure 7a**). Notice that the two intersections are different in nature: the one at ~1410 cm^{-1} is with the metallic substrate, leading to a reflection dip, while the resonance at ~1580 cm^{-1} is with the dielectric substrate, leading to a reflection peak (**Supplementary Figure 7b**). In comparison, the hBN/gold heterostructure with grating patterns only exhibits a single reflection dip with high-order modes with low amplitudes, and the suspended hBN grating shows a main reflection peak.

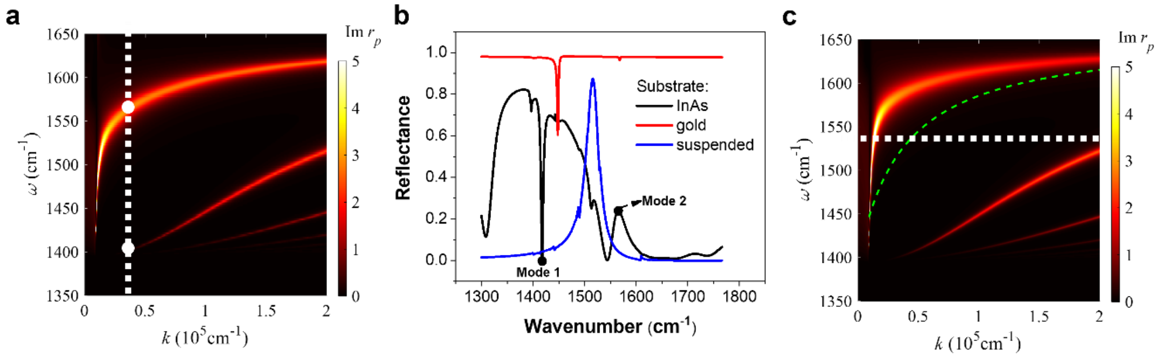

Supplementary Figure 7. HPhP dispersion of hBN/InAs with a modal transition and the implications. In the frequency domain. (a) The contour plot is the dispersion of hBN/InAs calculated by TMM. The white dashed reference line is the wavevector required for a hBN resonator with 450 nm width. (b) The simulated reflectance of hBN over different substrates. **In the real space.** (c) The contour plot is the dispersion of hBN/InAs calculated by TMM. The green curve is the dispersion of suspended hBN with the same thickness. The white reference line denotes the transitional frequency. The InAs discussed in this section has plasma frequency of 1613 cm-1.

In the near field, when HPhPs are propagating across hBN over different substrates, the propagation follows Snell's law. The k_{HPhP} of hBN/InAs are higher than suspended hBN below the transitional point, while the relationship is inverted after the transitional working frequency (**Supplementary Figure 7c**). If we consider a hBN over etched InAs heterostructure, the HPhPs will be guided in the high wavevector region, as discussed in our previous publication[1]. Therefore, below the transitional frequency (white reference line), the wavevector in hBN/InAs region will be higher than the suspended region, leading to guided HPhPs in

hBN/InAs. In contrast, above the transitional frequency, wavevectors of hBN/InAs will actually be smaller than suspended hBN (green dispersion curve), resulting in guided HPhPs in the suspended region.

Note 7. Data analysis on the transitional HPhP sample

Here we present the data analysis on the transitional HPhP sample, of which the InAs plasma frequency is ~1610 cm^{-1}. The s-SNOM amplitude images of this particular sample at different frequencies are shown in **Supplementary Figure 8c**. While polariton fringes are clearly observable at relatively low frequencies, e.g., 1442 and 1470 cm^{-1}, only "accumulated amplitude peak" is observed after the transitional frequency, similar to other high loss systems, e.g., monolayer hBN polaritons[2]. When the fringes are present, we employ the same strategy in **Supplementary Note 1** to extract the wavevectors. For frequencies above the transitional frequency, we follow the data analysis presented in Reference [3] to model the wavevector, and two representative fitted curves are shown in **Supplementary Figure 8a, b**.

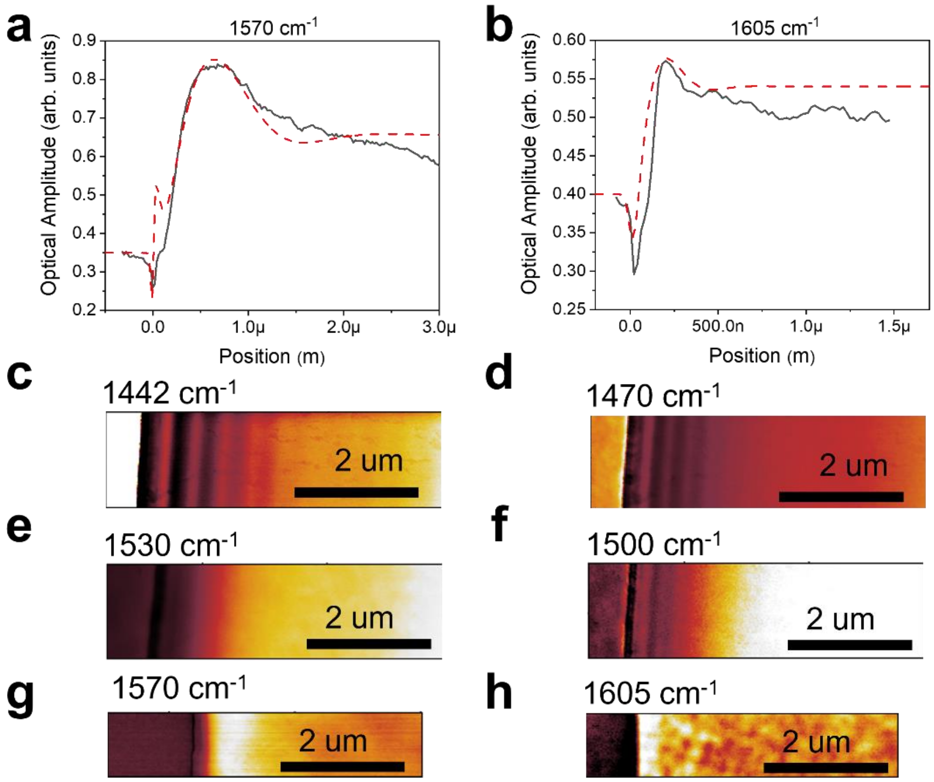

Supplementary Figure 8. Data analysis on the transitional HPhP sample. (a-b) Fitted optical amplitude plotted over the line profile acquired in s-SNOM images. (c-h) s-SNOM images at different frequencies. They are all at the same scale bar.

Note 8. Sensitivity to the local environment around modal order transition

Here we analyze the sensitivity of HPhPs to local environment, in a refractive index sensing context. We consider the dispersion of HPhPs of a structure: hBN over InAs, and the superstrate is with different permittivity values (1.0 and 1.1, respectively). The HPhPs change more significantly with only 0.1 permittivity change when it is on transitional InAs, and the transition frequency changes appreciably, as shown in **Supplementary Figure 9b**. In contrast, the HPhP dispersion change is less pronounced when the system is away from modal order transition points (**Supplementary Figure 9a, c**).

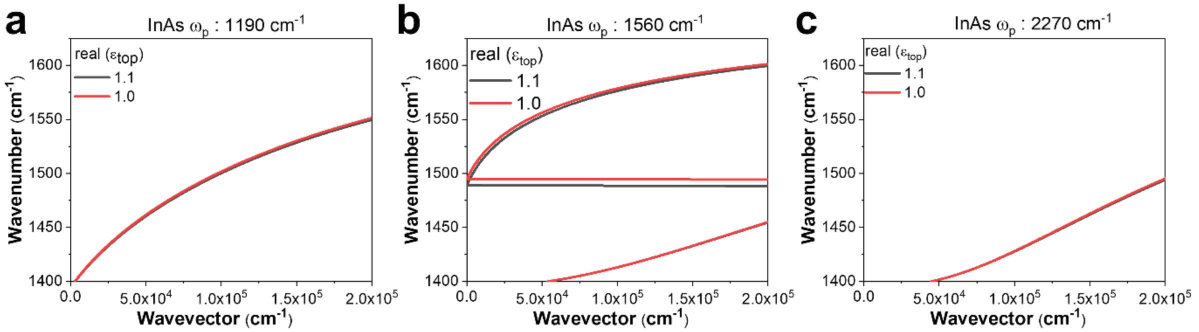

Supplementary Figure 9. HPhP dispersion of hBN/InAs with different InAs plasma frequency. The plasma frequency of InAs of panel a-c are 1190, 1560 and 2270 cm^{-1}, respectively.

Note 9. Loss associated with the tuned HPhPs

Here we discuss the loss of HPhPs as a function of InAs plasma frequency. In the calculation, we assumed a constant scattering rate of InAs and calculated the HPhP wavevectors and figure of merits (FOM). The FOM is defined as:

$$FOM = \frac{real(k_{HPhP})}{imag(k_{HPhP})}$$

While the wavevector contrast is maximized around the modal order transition, the FOM is compromised significantly (**Supplementary Figure 10**). Although the FOM is relatively low around the modal order transition, it is still mostly higher than the FOM of HPhPs supported by naturally abundant hBN and MoO_3 along the [100] axis (~20-30) allowing for HPhP applications where manipulation of the polariton response would prove critical.

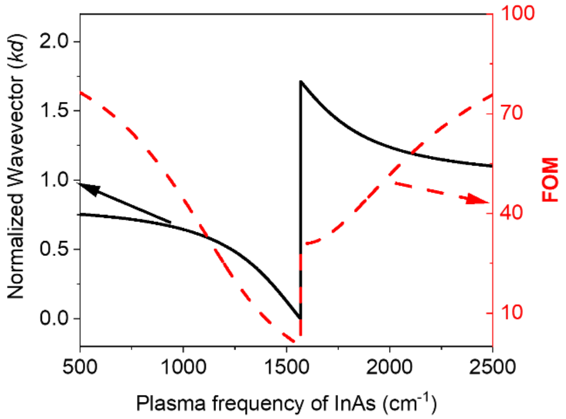

Supplementary Figure 10. HPhP FOM in a hBN/InAs heterostructure with varying InAs plasma frequency.

We further discuss a potential route to utilize the ultralow wavevector HPhPs without being affected by the associated loss: by using the hBN/InAs region as a cladding area to confine HPhP energy densities in a high wavevector region, e.g., a HPhP waveguide on suspended hBN, as shown in **Supplementary Figure 11b**. For a working frequency of 1500 cm^{-1}, hBN thickness of 100 nm, and InAs plasma frequency at 1500 cm^{-1}, the HPhP FOM on hBN/InAs area is only ~3, and the loss is too high to be useful. The k_{HPhP} is even lower than suspended hBN, indicating that a suspended hBN can serve as a “core material” to guide and confine HPhPs surrounded by hBN/InAs, with the concept being discussed thoroughly in our previous work[1]. With our previously developed analytical mode solution[1], we found that such a guided mode has a FOM of ~40, which is comparable to many HPhP systems. We followed the same simulation strategy[1] and verified the guided HPhPs in a suspended region (**Supplementary Figure 11a**).

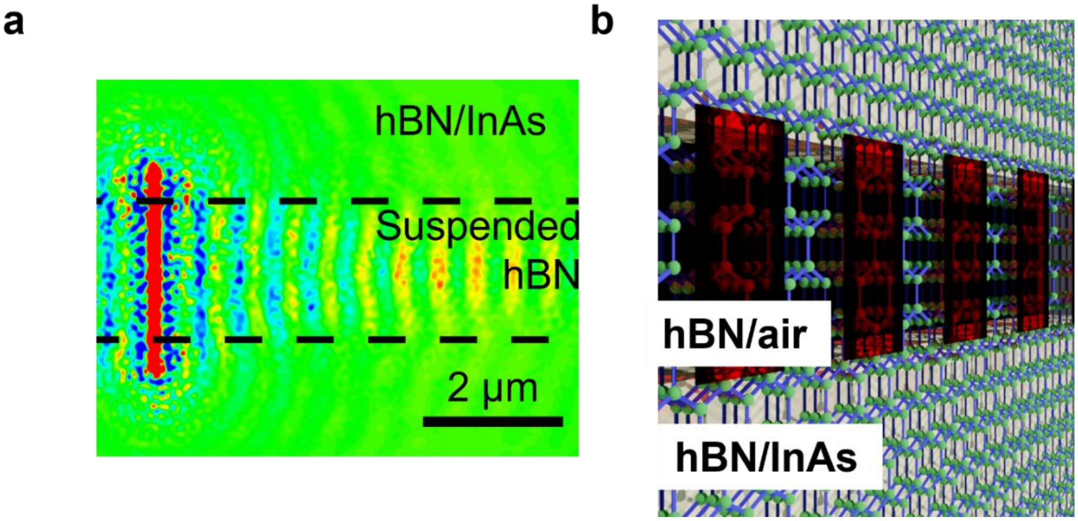

Supplementary Figure 11. Guided HPhPs on suspended hBN. a. Finite element simulation showing a guided mode in suspended hBN region (top view), with relatively long propagation length. The suspended hBN is surrounded by hBN/InAs. b. The schematic of the guided HPhP on suspended hBN to utilize the low wavevector HPhPs on hBN/InAs region.

Note 10. Dispersion of HPhPs at ultrafast time scale

Here we present the dispersion of HPhPs extracted from nano-FTIR at different time delays, as shown in **Supplementary Figure 12**. The data analysis is following reference [4]. The experimentally extracted dispersions are overlapped with analytically calculated dispersion at pumped (purple) and static (green) conditions.

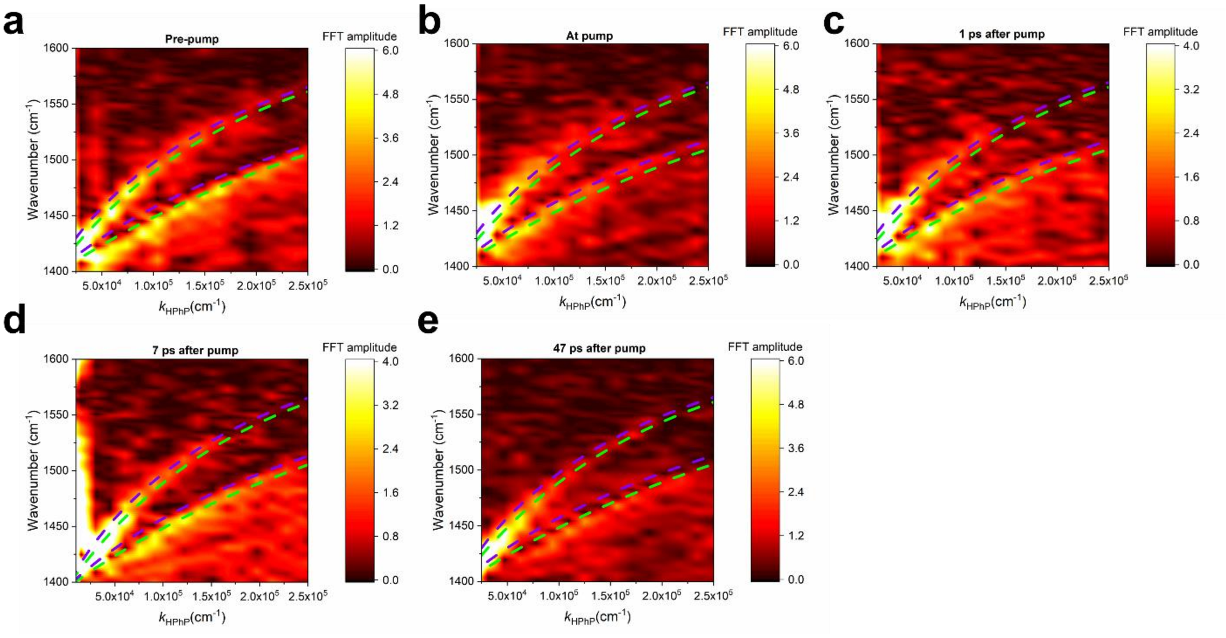

Supplementary Figure S12. Dispersions extracted from nano-FTIR scans. (a-e) Dispersions at different time delays. The analytical solutions are plotted with dashed curves. While the purple curves are calculated with InAs dielectric function at the pump arrival, the green curves are calculated with the static InAs dielectric function.

Note 11. Data process of ultrafast measurements

In the main text, **Fig. 4b,c**, we showed the nano-FTIR data at different time delays. Nano-FTIR data could feature multiple wrinkles due to laser stability issues, as shown in the raw data, **Supplementary Figure 13a**. We employed FFT filtering for every single nano-FTIR spectrum and replotted them, removing the artifacts from experiments, as shown in **Supplementary Figure 13b**. Note that the FFT filtering only

removes the artifacts without compromising the data, as the spectra shifting is observable with or without the FFT process.

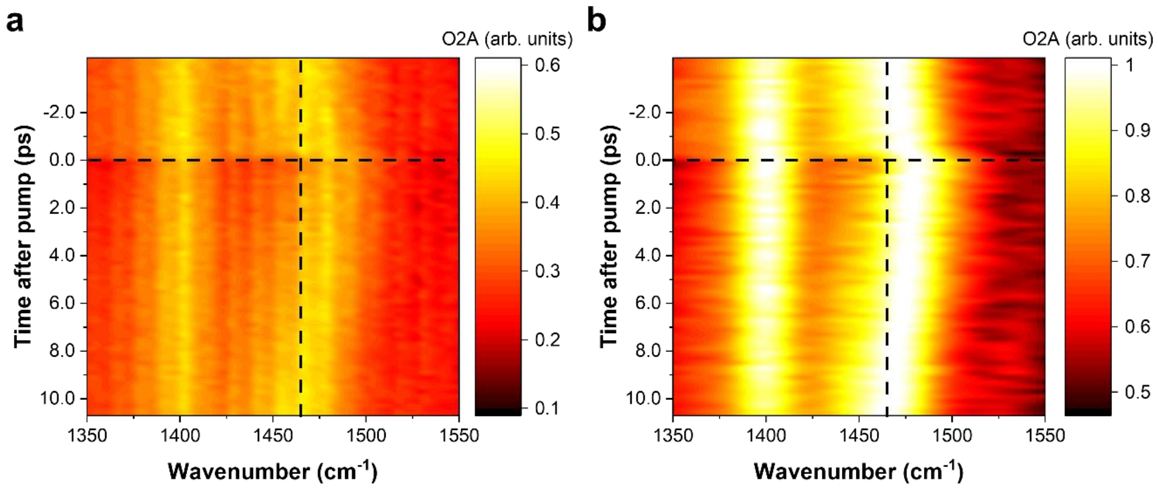

Supplementary Figure 13. FFT filtering of nano-FTIR optical amplitude. (a) Raw data of nano-FTIR at different time delays. (b) FFT filtered nano-FTIR data. (The two dashed reference lines show static polaritonic resonance frequency and pump arriving time, respectively.

Note 12. The collective effect of HPhPs in the temporal domain

To model the HPhPs in the temporal domain, we fitted the dielectric functions of InAs at different time delays with near-field data. The nano-FTIR spectra were calculated with a finite dipole model, and we consider the plasma frequency and scattering rate as fitting variables, with some representative fittings shown in **Supplementary Figure 14a-c**. The plasma frequencies at different time delays are then fitted with an exponential decay curve to find the carrier lifetimes of InAs, which is 8 ps (**Supplementary Figure 14d**).

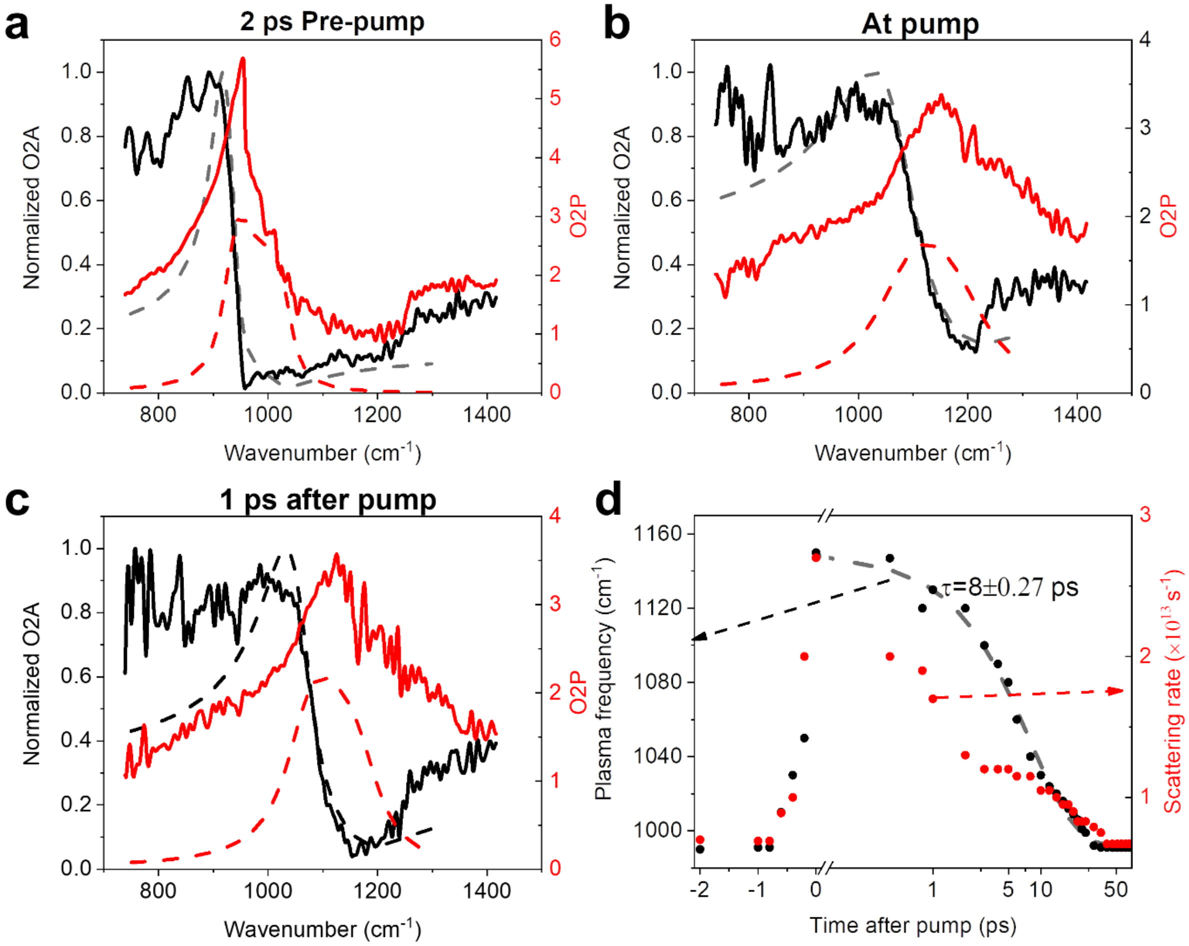

Supplementary Figure 14. Fitting of near-field InAs spectra. a-c, Measured near-field optical amplitude and phase at different time delays. The fittings were plotted with dashed curves. d, Plasma frequencies and scattering rate of InAs at different time delays. The plasma frequency is fitted with an exponential decay curve, and the resultant lifetime is 8 ps.

During the pump-probe measurement, the zero time delay is identified with an InAs reference, where the maximum optical amplitude is observed. For such a measurement, the probe beam is immediately reflected and then collected with the detector. However, for HPhP probes, when the probe beam arrives on the sample, the HPhPs will first be excited (at the probe arriving time), then it takes the HPhPs picoseconds to propagate before being scattered and detected, as shown in **Supplementary Figure 15**. Therefore, the HPhPs are actually influenced by InAs ω_p that is ~1-2 ps time after the probe beam arrives, which is a collective effect. With the HPhPs propagating, InAs plasma frequency is decreasing at picosecond time scales, leading to increasing polariton wavevectors, i.e., declining group velocity. Note that a static device highlighted by Feres et al[5] can realize polariton acceleration, and what is happening in our system is a decelerating polariton in the temporal domain.

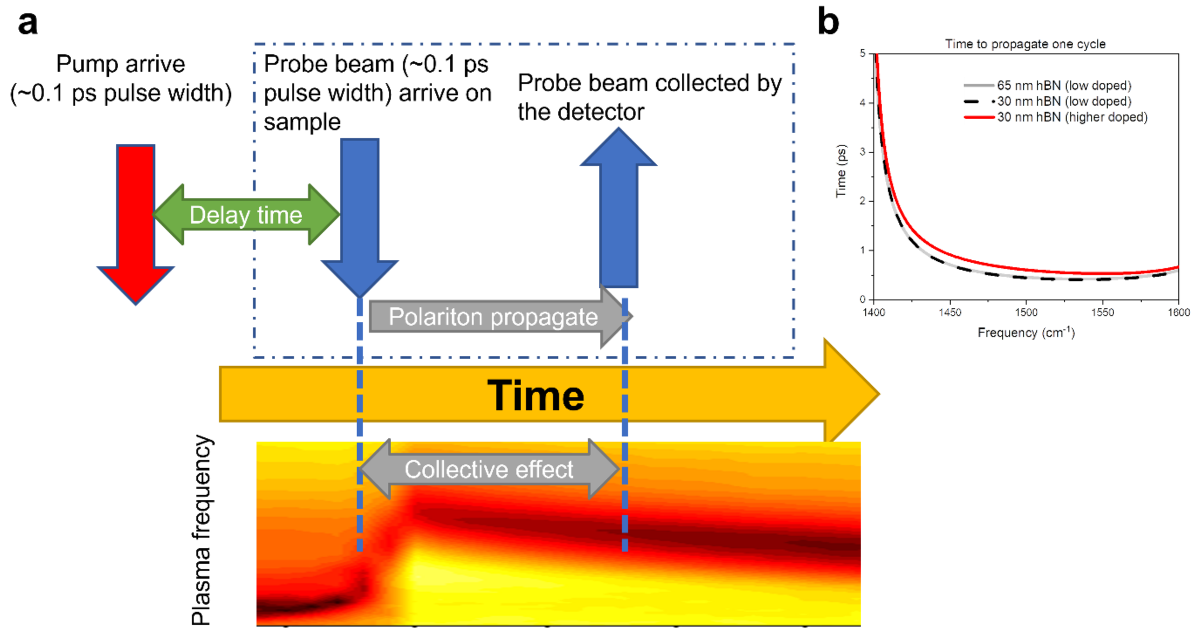

Supplementary Figure 15. Collective effects of polaritonic characteristics in the temporal domain. Schematic of the collective effect. In the pump-probe measurements, the delay time is defined as the difference between pump arrival and probe beam arrival. The InAs property is changing at picosecond time scale (**Supplementary Figure S14**). Thus, the polaritons experience decreasing plasma frequency during the propagation in the time domain. Note that the time scales in this panel are exaggerated to illustrate the concept, and they do not reflect the actual time scales. (b) Due to the low group velocity, the time for HPhPs to propagate 2π phase cycle is ~1 ps. The group velocity in this panel was calculated, while one could directly extract the group velocity following the procedures described in references[6,7].

All the above discussions assume a uniform InAs pump, and our system can be approximated in such a way.

We first discuss the beam profile in the x-y plane. The pump laser (1560 nm wavelength) is focused by an off-axis parabolic mirror (NA=0.7). Because of the limited beam diameter of the pump beam (~2-3 mm) and much larger focal length (larger than 10 mm), the focal spot is relatively large (at least 3-5λ, i.e., 5-8 μm). Due to the challenge of the alignment, the actual focal spot is even larger than the calculated optimal condition. In this case, the power variation within the collected region (~ 2 um) is below 10% and can be neglected, and the InAs can be approximately considered uniform pumped.

We then discuss the absorbed energy at different depths, since the absorbed energy (i.e., the excited carrier concentration) decays when the pump laser penetrates into the InAs material. For this purpose, we calculated the absorption at different depths using a layer-resolved absorption calculation[8], and the absorption at 100 nm below the surface is only decreased by ~8% as compared to the top surface. As both

nano-FTIR measurements in Fig. 4c and HPhP are dominated by the ~100 nm InAs below the surface[9,10], the carrier concentration variation in the z-axis can be neglected.

Due to the collective effect, the change of HPhP wavevectors will be within the shaded bands (**Supplementary Figure 16**), which denote the plasma frequency of InAs between 0 ps and 2 ps time delay. We approximate the change by taking the point in the center. As indicated by the calculation, the wavevector change at ~ 1450 cm^{-1} should be ~1.2 times, which agrees with our experimental data very well (λ_{HPhP} change from 1.15 µm to 1.37 µm, 1.19 times difference).

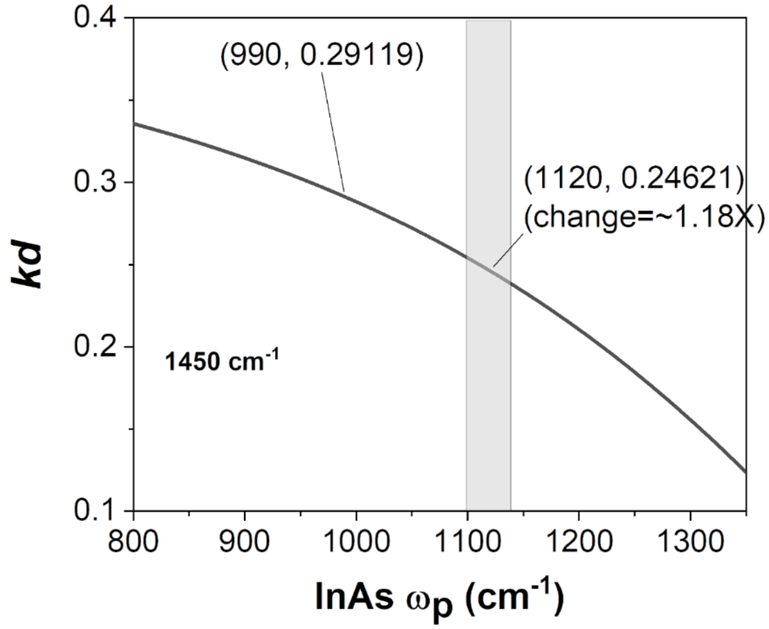

Supplementary Figure 16. Collective effects of polaritonic characteristics in the temporal domain. The normalized HPhP values at 1450 cm^{-1} and 1500 cm^{-1} with InAs of different plasma frequencies. The plasma frequency of static InAs is approximately 990 cm^{-1}, while the pumped InAs at 0-2 ps after pump features plasma frequency in the shaded range.

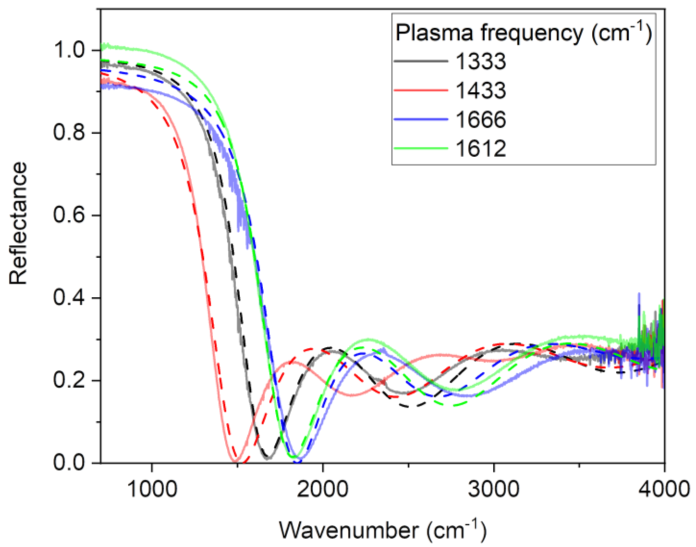

Supplementary Figure 17. The fitting of FTIR data to determine plasma frequencies of InAs. The dashed curves were calculated via the transfer matrix method while the solid curves were experimental data.

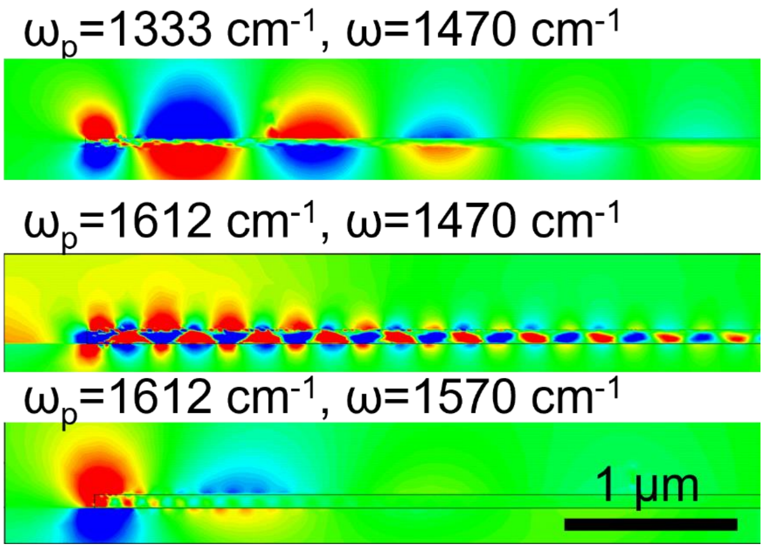

Supplementary Figure 18. The cross-sectional field profiles of hBN/InAs heterostructures. The simulation parameters (frequency, thickness and InAs ω_p) are identical to Fig. 3d. The polaritonic wavelength tuning is through the interaction between the evanescent field of HPhPs and the substrate.

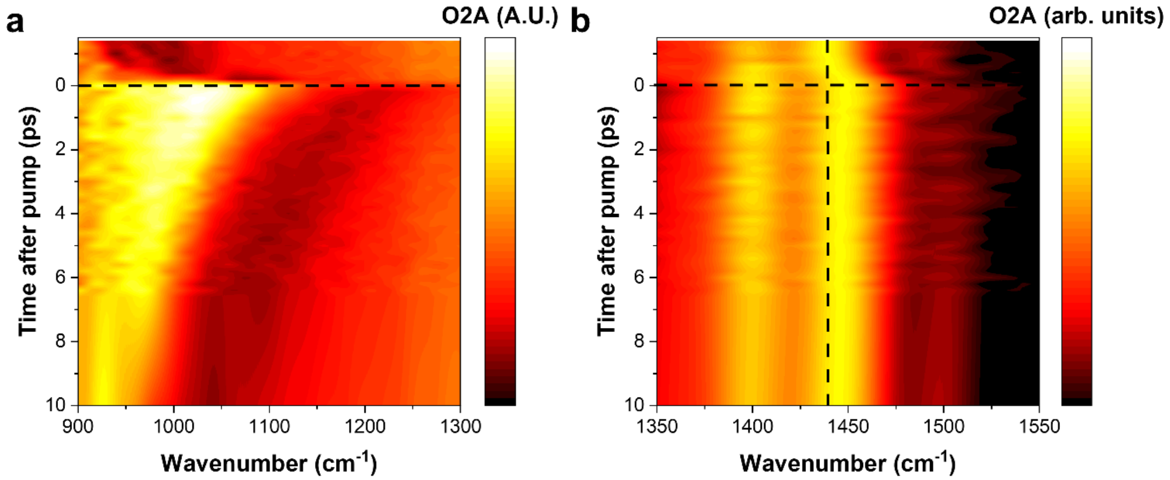

Supplementary Figure 19. Another measurement similar to Fig.4b but at a different spatial location. Similar measurements compared to the data in Fig. 4b in the main text. The measurement was performed at different spatial location (~0.5 μm) from the edge of the same hBN, and the modulation characteristics are very similar to the data in the main text: the HPhP frequency is increased within 1 ps and the lifetime is about 8 ps.

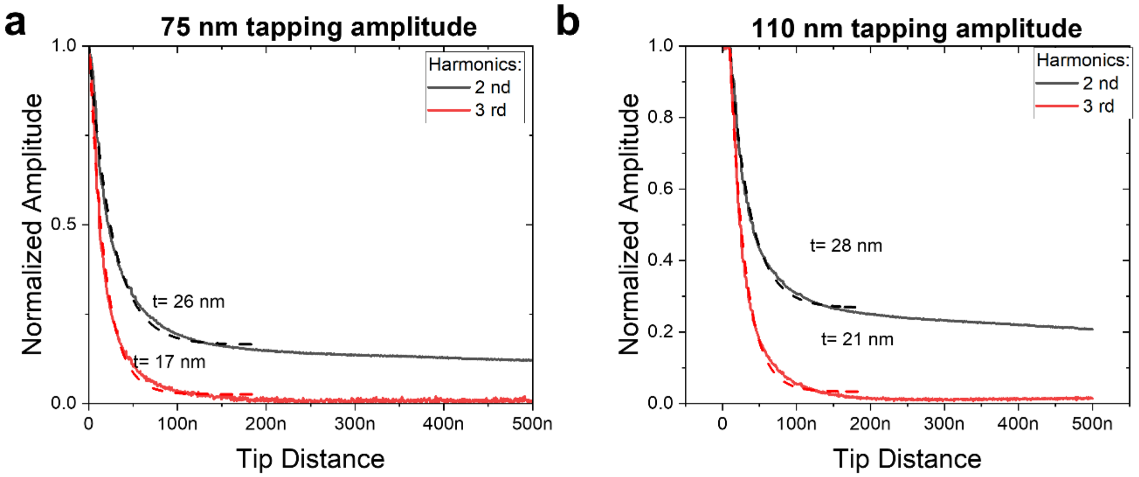

Supplementary Figure

20. The approach curves for 2nd and 3rd harmonic signals for different tapping amplitude. In both cases, the 3rd harmonic signals are dominated by the near-field contribution (the signal exponentially decays if the tip-sample distance is over ~20 nm (*t* in the figure)).

Supplementary Table 1. Strategies of tuning and modulating HPhPs

Tuning strategy	Reference	Tuning range	Speed if dynamic
Structuring of hyperbolic media	[11,12]	N.A.<br>(Highlight structured HPhP instead of wavevector control)	Static
Substrate tuning with phase change	[13-15]	~60%	Nanoseconds
Substrate tuning with graphene	[16-21]	~20% experimental[16] and ~100% simulation	Not characterized. Could be picosecond level
Tuning with static substrate	[14,15,22-25]	~400%	Static
Substrate tuning with static doped semiconductor	This work	~800%	Static
Optically pumped doped semiconductor	This work	~20%	Picoseconds

Supplementary References.

1. He, M. *et al.* Guided Mid‐IR and Near‐IR Light within a Hybrid Hyperbolic‐Material/Silicon Waveguide Heterostructure. *Adv Mater* **33**, 2004305 (2021).
2. Dai, S. *et al.* Phonon polaritons in monolayers of hexagonal boron nitride. *Adv Mater* **31**, 1806603 (2019).
3. Gerber, J. A., Berweger, S., O'Callahan, B. T. & Raschke, M. B. Phase-resolved surface plasmon interferometry of graphene. *Physical review letters* **113**, 055502 (2014).
4. Bylinkin, A. *et al.* Real-space observation of vibrational strong coupling between propagating phonon polaritons and organic molecules. *Nat Photonics*, 1-6 (2020).
5. Feres, F. H., Mayer, R. A., Barcelos, I. D., Freitas, R. O. & Maia, F. C. B. Acceleration of subwavelength polaritons by engineering dielectric-metallic substrates. *Acs Photonics* **7**, 1396-1402 (2020).
6. Yoxall, E. *et al.* Direct observation of ultraslow hyperbolic polariton propagation with negative phase velocity. *Nat Photonics* **9**, 674-678 (2015).
7. Zhang, X. *et al.* Ultrafast anisotropic dynamics of hyperbolic nanolight pulse propagation. *Sci Adv* **9**, eadi4407 (2023).
8. Passler, N. C., Jeannin, M. & Paarmann, A. Layer-resolved absorption of light in arbitrarily anisotropic heterostructures. *Physical Review B* **101**, 165425 (2020).
9. Govyadinov, A. A. *et al.* Recovery of permittivity and depth from near-field data as a step toward infrared nanotomography. *Acs Nano* **8**, 6911-6921 (2014).
10. Lee, I.-H. *et al.* Image polaritons in boron nitride for extreme polariton confinement with low losses. *Nature communications* **11**, 1-8 (2020).
11. Dai, Z. *et al.* Edge-oriented and steerable hyperbolic polaritons in anisotropic van der Waals nanocavities. *Nature communications* **11**, 1-8 (2020).
12. Herzig Sheinfux, H. *et al.* Transverse Hypercrystals Formed by Periodically Modulated Phonon Polaritons. *ACS Nano* **17**, 7377-7383 (2023).
13. Chaudhary, K. *et al.* Polariton nanophotonics using phase-change materials. *Nature communications* **10**, 1-6 (2019).
14. Folland, T. G. *et al.* Reconfigurable infrared hyperbolic metasurfaces using phase change materials. *Nature Communications* **9**, 4371, doi:10.1038/s41467-018-06858-y (2018).
15. Dai, S. *et al.* Phase‐Change Hyperbolic Heterostructures for Nanopolaritonics: A Case Study of hBN/VO2. *Adv Mater*, 1900251 (2019).
16. Dai, S. *et al.* Graphene on hexagonal boron nitride as a tunable hyperbolic metamaterial. *Nature nanotechnology* **10**, 682-686 (2015).
17. Álvarez-Pérez, G. *et al.* Active tuning of highly anisotropic phonon polaritons in van der Waals crystal slabs by gated graphene. *Acs Photonics* **9**, 383-390 (2022).
18. Zeng, Y. *et al.* Tailoring topological transitions of anisotropic polaritons by interface engineering in biaxial crystals. *Nano Letters* **22**, 4260-4268 (2022).
19. Ruta, F. L. *et al.* Surface plasmons induce topological transition in graphene/α-MoO3 heterostructures. *Nature communications* **13**, 1-7 (2022).
20. Hu, H. *et al.* Gate-tunable negative refraction of mid-infrared polaritons. *Science* **379**, 558-561 (2023).

21 Hu, H. *et al.* Doping-driven topological polaritons in graphene/α-MoO3 heterostructures. *Nature Nanotechnology* **17**, 940-946 (2022).
22 Fali, A. *et al.* Refractive Index-Based Control of Hyperbolic Phonon-Polariton Propagation. *Nano letters* **19**, 7725-7734 (2019).
23 Ambrosio, A. *et al.* Selective excitation and imaging of ultraslow phonon polaritons in thin hexagonal boron nitride crystals. *Light: Science & Applications* **7**, 1-9 (2018).
24 Kim, K. S. *et al.* The Effect of Adjacent Materials on the Propagation of Phonon Polaritons in Hexagonal Boron Nitride. *The Journal of Physical Chemistry Letters* **8**, 2902-2908, doi:10.1021/acs.jpclett.7b01048 (2017).
25 Shen, J. *et al.* Hyperbolic phonon polaritons with positive and negative phase velocities in suspended α-MoO3. *Applied Physics Letters* **120**, 113101 (2022).
